# Co-Metabolic Degradation of β-Cypermethrin and 3-Phenoxybenzoic Acid by Co-Culture of *Bacillus licheniformis* B-1 and *Aspergillus oryzae* M-4

**DOI:** 10.1371/journal.pone.0166796

**Published:** 2016-11-29

**Authors:** Jiayuan Zhao, Yuanlong Chi, Yingchao Xu, Dongying Jia, Kai Yao

**Affiliations:** College of Light Industry, Textile & Food Engineering, Sichuan University, 610065, Chengdu, Sichuan, P. R. China; MJP Rohilkhand University, INDIA

## Abstract

The degradation efficiency of organic contaminants and their associated metabolites by co-culture of microbes is mainly limited by toxic intermediates from co-metabolic degradation. In this study, we investigated the degradation of β-cypermethrin (β-CY) and 3-phenoxybenzoic acid (3-PBA) by co-culture of *Bacillus licheniformis* B-1 and *Aspergillus oryzae* M-4, as well as the influences of β-CY and 3-PBA metabolites on their degradation and the growth of strains B-1 and M-4. Our results indicated that 100 mg/L β-CY was degraded by 78.85%, and 3-PBA concentration was 0.05 mg/L after 72 h. Compared with using only strain B-1, the half-life (*t*_1/2_) of β-CY by using the two strains together was shortened from 84.53 h to 38.54 h, and the yield coefficient of 3-PBA was decreased from 0.846 to 0.001. At 100 mg/L of 3-PBA and gallic acid, β-CY and 3-PBA degradation were only 17.68% and 40.45%, respectively. As the toxic intermediate derived from co-metabolic degradation of β-CY by strain B-1, 3-PBA was efficiently degraded by strain M-4, and gallic acid, as the toxic intermediate from co-metabolic degradation of 3-PBA by strain M-4, was efficiently degraded by strain B-1. These results provided a promising approach for efficient biodegradation of β-CY and 3-PBA.

## Introduction

Pyrethroid pesticides are the most widely used insecticides, and their residues are consistently detected in fruits and vegetables, rivers, and soil as a result of excessive spraying [1, 2]. Hladik et al. [3] reported that the concentrations of pyrethroids in bed sediments from urban and agricultural streams across the United States ranged from 0.3 to 180 ng/g dry weight. Beta-cypermethrin (β-CY) is an important pyrethroid pesticide, its residue can accumulate in the human body through the food supply chain and exhibit toxic effects on the human reproductive, immune, and nervous systems [4–6]. As a β-CY metabolite, 3-phenoxybenzoic acid (3-PBA) is difficult to degrade further in the environment due to its diphenyl oxide structure [7]. Heudorf et al. [8] reported that 3-PBA residue in urine of children and adolescents in Germany was 0.30 μg/L. 3-PBA can also be toxic to humans through disruption of the normal secretion of reproductive hormones and breakage of sperm DNA, which lowers sperm count [9–11]. Therefore, it is critically necessary to degrade β-CY and 3-PBA.

As organic contaminants, pyrethroid and 3-PBA residues are generally removed using microbial methods [12–14], where the co-metabolism is the main process for contaminant removal [15, 16]. Co-metabolic biodegradation is defined as the transformation of a non-growth substrate in the obligatory presence of a growth substrate or another utilizable compound [15, 16]. Compared with other methods, co-metabolic biodegradation of organic contaminants presents several important advantages, such as higher degradation rates and applicability [17, 18]. However, some reports verified that microbial co-metabolic degradation of organic contaminants generated toxic intermediates that significantly inhibited the degradation of parent compounds and microbial growth [15–18]. Recently, Han Tran et al. [16] proved that the co-culture of microbes could efficiently degrade organic contaminants and associated metabolites via co-metabolism. However, to the best of our knowledge, no research has been studied on the effects of intermediates from co-metabolic degradation of β-CY and 3-PBA on their degradation efficiency by microbial co-culture so far.

*Bacillus licheniformis* B-1 is capable of efficiently degrading β-CY via co-metabolism, but was unable to degrade 3-PBA [13]. Previous reports demonstrated that fungi are more efficient than bacteria in degrading compounds with benzene ring structures [15,16,19], implying that fungi should be more suitable to degrade 3-PBA as compared to bacteria [19]. *Aspergillus oryzae* M-4 was obtained from soy sauce koji and is capable of degrading 3-PBA via co-metabolic biodegradation, but was unable to degrade β-CY [20].

In this study, we investigated the degradation of β-CY and 3-PBA by co-culture of *B*. *licheniformis* B-1 and *A*. *oryzae* M-4, as well as the effects of their intermediate-metabolites on their degradation and the growth of strain B-1 (or strain M-4). Our results suggested that this approach is capable of efficiently degrading pyrethroids and 3-PBA, and we elucidated the possible mechanisms involved in the efficient degradation of organic contaminants and their associated metabolites via co-metabolism.

## Materials and Methods

### Materials

β-CY (99.7%) and 3-PBA (98%) were obtained from the National Standard Substances Center (Beijing, China) and Sigma-Aldrich Chemical Co. (Shanghai, China), respectively. Chromatographic-grade acetonitrile was purchased from Tedia Co. (Fairfield, OH., USA). Acetonitrile, ethyl alcohol, KH_2_PO_4_, K_2_HPO_4,_ MgSO_4_, NaCl, NaOH, Na_2_SO_4_, (NH_4_)_2_SO_4_, phenol, catechol, and gallic acid were of analytical grade and procured from Kelong Chemical Co. (Chengdu, China).

### Microorganisms and media

*B*. *licheniformis* B-1 was isolated from the soil in a tea garden (Ya’an, China) and was capable of transforming β-CY into 3-PBA and chrysanthemic acid, but was unable to degrade 3-PBA. Strain B-1 could degrade 46.54% of β-CY (100 mg/L) at 30°C and shaking at 180 rpm in Luria-Bertani and mineral salt (LB-MS) media after incubation for 72 h. *A*. *oryzae* M-4 was obtained from soy sauce koji and was capable of transforming 3-PBA into phenol and gallic acid, with a further transformation of phenol into catechol. Strain M-4 degraded 62.76% of 3-PBA (100 mg/L) at 30°C and shaking at 180 rpm in LB-MS media after incubation for 72 h, but was unable to degrade β-CY.

LB-MS medium was prepared according to previously described methods [13] and consisted of LB and MS at a ratio of 2:1 (v/v). The pH value of medium was adjusted to between 7.0 and 7.5 prior to sterilization at 121°C for 20 min.

### Inoculum preparation

The inoculum of strain B-1 was prepared according to previously reported methods [14]. The spores of strain M-4 were transferred and suspended in normal saline (0.9% NaCl) to achieve an absorbance of about 0.15 at 490 nm. The resulting fungal spore suspension was used as the inoculum of strain M-4.

### Determination of β-CY and 3-PBA concentration

Thirty milliliters of media and acetonitrile were transferred into a 100-mL Erlenmeyer flask and shaken for 30 s by a vortex mixer. The flask was then subjected to ultrasonication (40 kHz and 300 W) for 30 min. After the mixture was centrifuged at 8000 rpm for 20 min, the supernatant was collected and filtered through a 0.22-μm membrane filter [21]. The concentrations of β-CY and 3-PBA were determined according to previously described methods [13], and residual substrate (%) and degradation (%) were calculated according to the eqs (1) and (2), respectively.

$$\text{Residual substrate}\left(\text{\%}\right)\;=\left(C_{K}/C\right)\times 100$$ (1)

$$\text{Degradation}\left(\text{\%}\right)\;=\left(1-C_{K}/C\right)\times 100$$ (2)

where *C*_k_ is the residual concentration of β-CY or 3-PBA in the sample solution (mg/L) and *C* is the initial concentrations of β-CY or 3-PBA (mg/L) which is measured at time zero.

### β-CY and 3-PBA degradation by co-culture of strains B-1 and M-4

Thirty milliliters of LB-MS medium containing 100 mg/L of β-CY was mixed with 1 mL of strain B-1 inoculum and 0.5 mL of strain M-4 inoculum and incubated with shaking at 180 rpm and 30°C. LB-MS medium with 100 mg/L of β-CY and 1.5 mL of strain B-1 inoculum was served as a control. The residual β-CY (%) and the concentrations (mg/L) of 3-PBA in the medium were measured every 12 h, and the degradation rate constant (*k*), half-life (*t*_1/2_), and yield coefficient (*Y*) were calculated according to previously described methods [13].

### Effects of intermediate metabolites on microbial biomass and β-CY or 3-PBA degradation

Various concentrations (20, 40, 60, 80, and 100 mg/L) of 3-PBA, chrysanthemic acid, gallic acid, phenol, or catechol were dissolved in 30 mL of LB-MS medium with 100 mg/L β-CY (or 3-PBA), followed by addition of 1.5 mL of strain B-1 (or strain M-4) inoculum. All cultures were incubated with shaking at 180 rpm and 30°C for 72 h. The sample without β-CY and 3-PBA metabolites was served as a control. The concentrations (mg/L) of β-CY or 3-PBA and the biomass (OD_600_) of strain B-1 or the dry cell weight of strain M-4 (g/L) were measured, and β-CY or 3-PBA degradation (%) was calculated. The biomass of strain B-1 was determined at OD_600_. Dry cell weight (g/L) was obtained using filtering media and drying at 80°C and represented the biomass of strain M-4. The metabolites were detected according to a previously reported method [19], and the final concentration (mg/L) was calculated according to the following equation:

$$\text{Final concentration}\left(\text{mg/L}\right)\;=C_{\text{f}}-C_{0}$$ (3)

where *C*_f_ and *C*_0_ are the residual concentrations of β-CY or 3-PBA metabolites in the sample and control solution (mg/L), respectively.

### Statistical analysis

Each experiment was performed in triplicate, and the results were expressed as the means of three replicates with standard deviations. All statistical analyses were performed using SPSS version 17.0 (SPSS Inc., Chicago, IL, USA).

## Results

### Co-metabolic degradation of β-CY and 3-PBA by co-culture of strains B-1 and M-4

**Fig 1:**
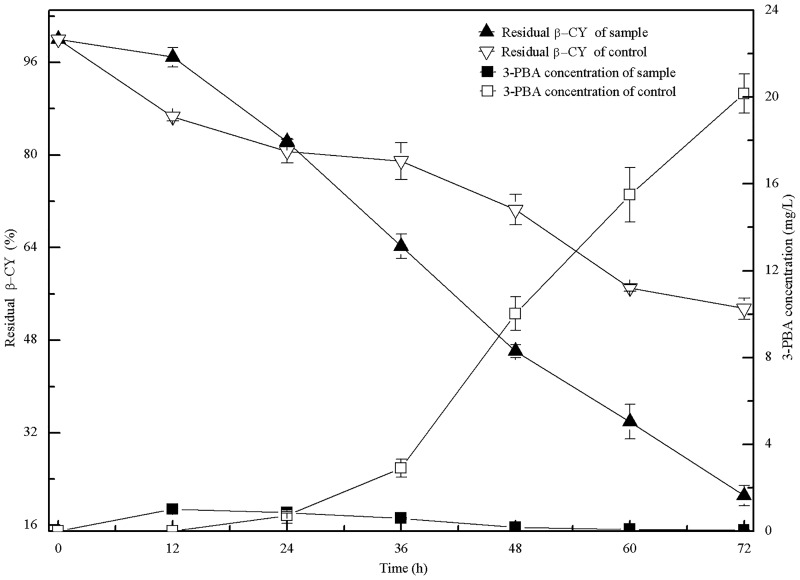
Residual β-CY and 3-PBA concentration after incubation with strain B-1 alone (control) or co-culture with strain B-1 and strain M-4 (sample). Triangles represent residual β-CY. Squares represent 3-PBA concentration.

The residual β-CY and the concentration of 3-PBA by co-culture of strains B-1 and M-4 during the incubation period are shown in Fig 1. Co-culture of strains B-1 and M-4 was more efficient in degrading β-CY than culture of strain B-1 alone. At 72 h, residual β-CY was 21.15% by co-culture of strains B-1 and M-4, almost cutting 50% off that observed using only strain B-1. Additionally, because strain B-1 was unable to degrade 3-PBA, 3-PBA concentration increased along with the time period, reaching a maximum concentration of 20.16 mg/L. And 3-PBA was efficient degraded by co-culture of the two strains, with its final concentration at near zero.

First-order kinetic parameters for the degradation of 100 mg/L β-CY by strain B-1 alone and by co-culture of strains B-1 and M-4 after incubation for 72 h are presented separately in Table 1. Compared with the inoculation with strain B-1, the half-life (*t*_1/2_) of β-CY during co-culture of the two strains was reduced 53.22%, 3-PBA yield coefficient (*Y*) decreasing from 0.846 to 0.001 (Table 1). Therefore, these results indicated that co-culture of the two strains degraded β-CY efficiently and 3-PBA completely.

**Table 1:** First-order kinetic parameters of β-CY degradation by strain B-1 alone or by co-culture with strains B-1 and M-4 after incubation for 72 h.

Treatment	Regression equation	*k* (h^−1^)	*t*_1/2_ (h)	*R*^*2*^	Y
Strain B-1	*C*_*t*_ = 99.2857*e*^−0.008*t*^	0.008	84.53	0.950	0.846
Strains B-1 and M-4	*C*_*t*_ = 110.1034*e*^−0.018*t*^	0.018	39.54	0.914	0.001

### Toxic intermediates from co-metabolic degradation of β-CY

**Fig 2:**
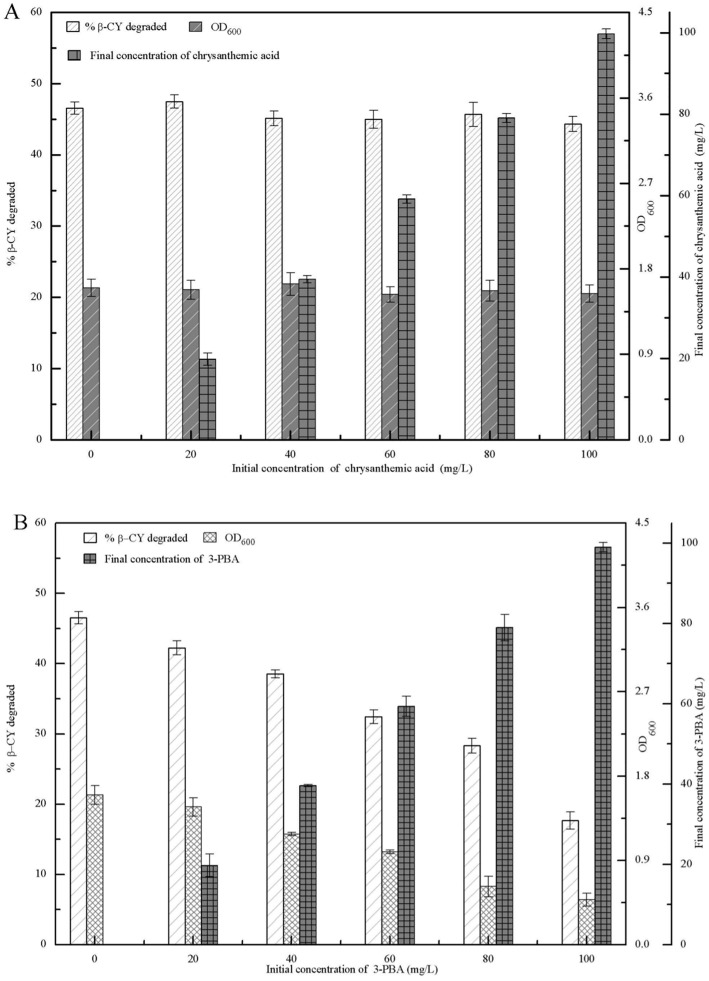
β-CY degradation, biomass (OD_600_) of strain B-1, and the final β-CY-metabolite concentration in LB-MS media supplemented with- chrysanthemic acid or - 3-PBA after 72 h. (A) chrysanthemic acid. (B) 3-PBA.

The β-CY degradation, the concentrations of β-CY metabolites, and the biomass (OD_600_) of strain B-1 were determined in the presence of various concentrations of chrysanthemic acid and 3-PBA after incubation for 72 h. As shown in Fig 2A, the β-CY degradation and the biomass of strain B-1 remained unchanged after addition of chrysanthemic acid. Compared with the initial concentrations of chrysanthemic acid, the final concentrations were almost unchanged, suggesting that chrysanthemic acid could not be used by strain B-1 as a carbon or energy source for promoting β-CY degradation and the growth of strain B-1. Additionally, after the addition of 3-PBA, both β-CY degradation and the biomass of strain B-1 decreased in the presence of increasing 3-PBA concentrations (Fig 2B). The final concentration of 3-PBA exhibited a minimal decrease when compared with the initial concentration, indicating that 3-PBA could not be utilized by strain B-1. Furthermore, 3-PBA inhibited β-CY degradation and decreased the biomass of strain B-1. These results suggested that 3-PBA was the toxic intermediate generated by co-metabolic degradation of β-CY.

### Toxic intermediates from co-metabolic degradation of 3-PBA

**Fig 3:**
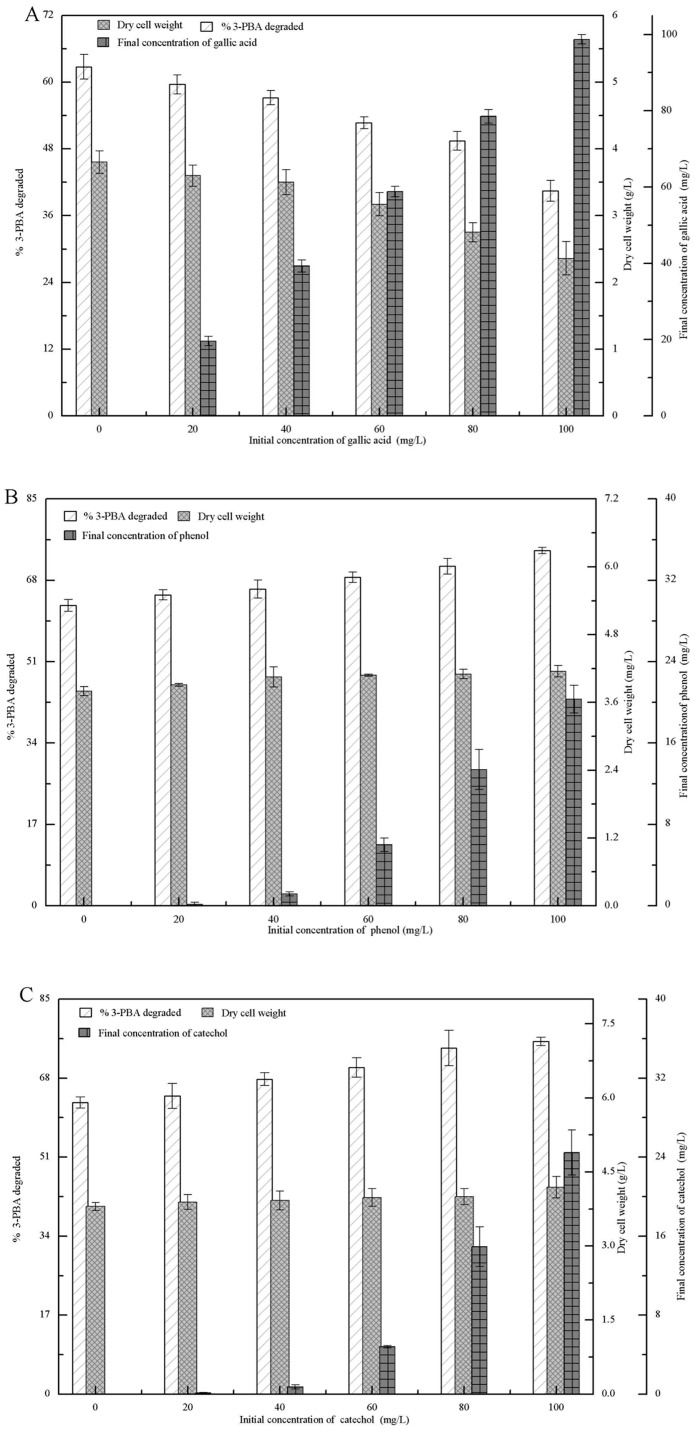
3-PBA degradation, the dry cell weight of strain M-4, and the final 3-PBA-metabolite concentration in LB-MS media supplemented with gallic acid, phenol, or catechol after 72 h. (A) gallic acid. (B) phenol. (C) catechol.

3-PBA degradatio, 3-PBA-metabolite concentrations, and the biomass (dry cell weight, g/L) of strain M-4 were investigated in the presence of various concentrations of gallic acid, phenol, and catechol after incubation for 72 h. As shown in Fig 3A, after the addition of gallic acid, the initial concentrations of gallic acid were negatively correlated with 3-PBA degradation and the biomass of strain M-4, and the final gallic acid concentrations were almost equal to the initial concentrations. Our results indicated that gallic acid was not utilized by strain M-4, and gallic acid inhibited cell growth and 3-PBA degradation. After the addition of phenol and catechol, Fig 3B and 3C show that the final concentrations of phenol and catechol were significantly less than their initial concentrations, and the biomass of strain M-4 and 3-PBA degradation increased along with their initial concentrations increased. These results suggested that these two compounds could be utilized by strain M-4 for 3-PBA degradation and cell growth, and that gallic acid was the toxic intermediate from co-metabolic degradation of 3-PBA.

### Effect of β-CY metabolites on 3-PBA degradation

**Fig 4:**
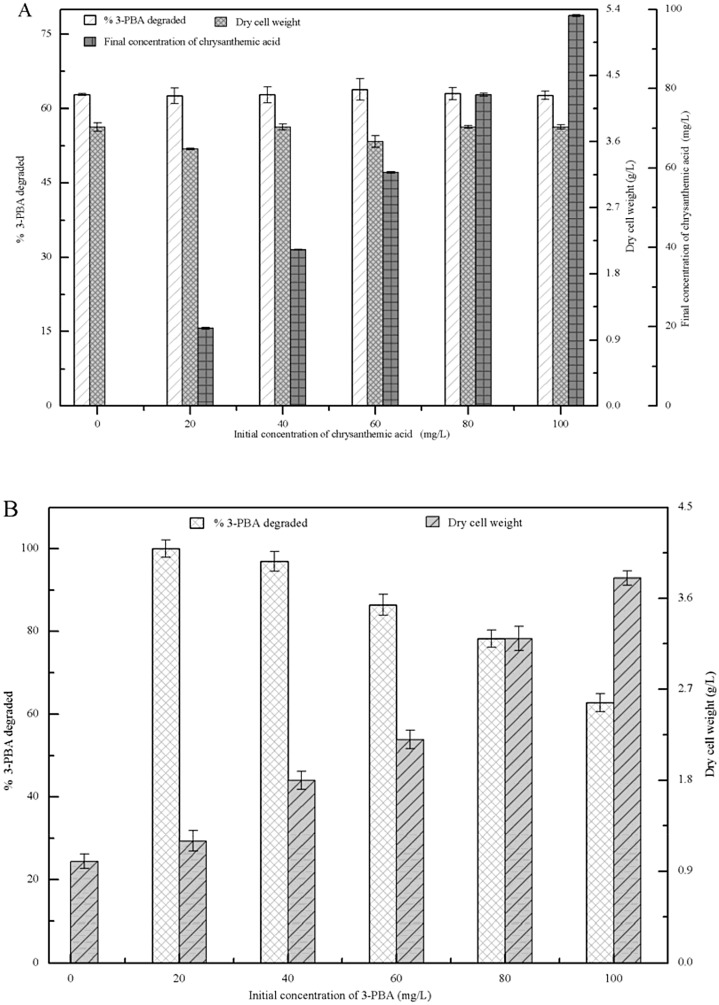
3-PBA degradation and the biomass of strain M-4 in LB-MS media supplemented with chrysanthemic acid or 3-PBA after 72 h. (A) chrysanthemic acid-. (B) 3-PBA.

3-PBA degradation, β-CY-metabolite concentrations, and the biomass of strain M-4 were determined in the presence of various concentrations of chrysanthemic acid and 3-PBA after incubation for 72 h. As shown in Fig 4A, when the initial concentrations of chrysanthemic acid ranged from 20 mg/L to 100 mg/L, its final concentrations did not noticeably decrease, and both the dry cell weight of strain M-4 and 3-PBA degradation were unchanged after incubation for 72 h. These results demonstrated that chrysanthemic acid was not consumed by strain M-4 to promote 3-PBA degradation and cell growth. Fig 4B shows that when the initial concentrations of 3-PBA were below 40 mg/L, its degradation was almost 100%. Even when the initial concentration reached 100 mg/L, 62.76% of 3-PBA was degraded by strain M-4. Additionally, the dry cell weight of strain M-4 increased from 1.2 g/L to 3.8 g/L when the initial concentrations of 3-PBA rose from 20 mg/L to 100 mg/L. These results indicated that 3-PBA improved the growth of strain M-4 and that chrysanthemic acid did not affect 3-PBA degradation by this strain. Furthermore, 3-PBA, the toxic intermediate from co-metabolic degradation of β-CY, was efficiently degraded by strain M-4.

### Effect of 3-PBA metabolites on β-CY degradation

**Fig 5:**
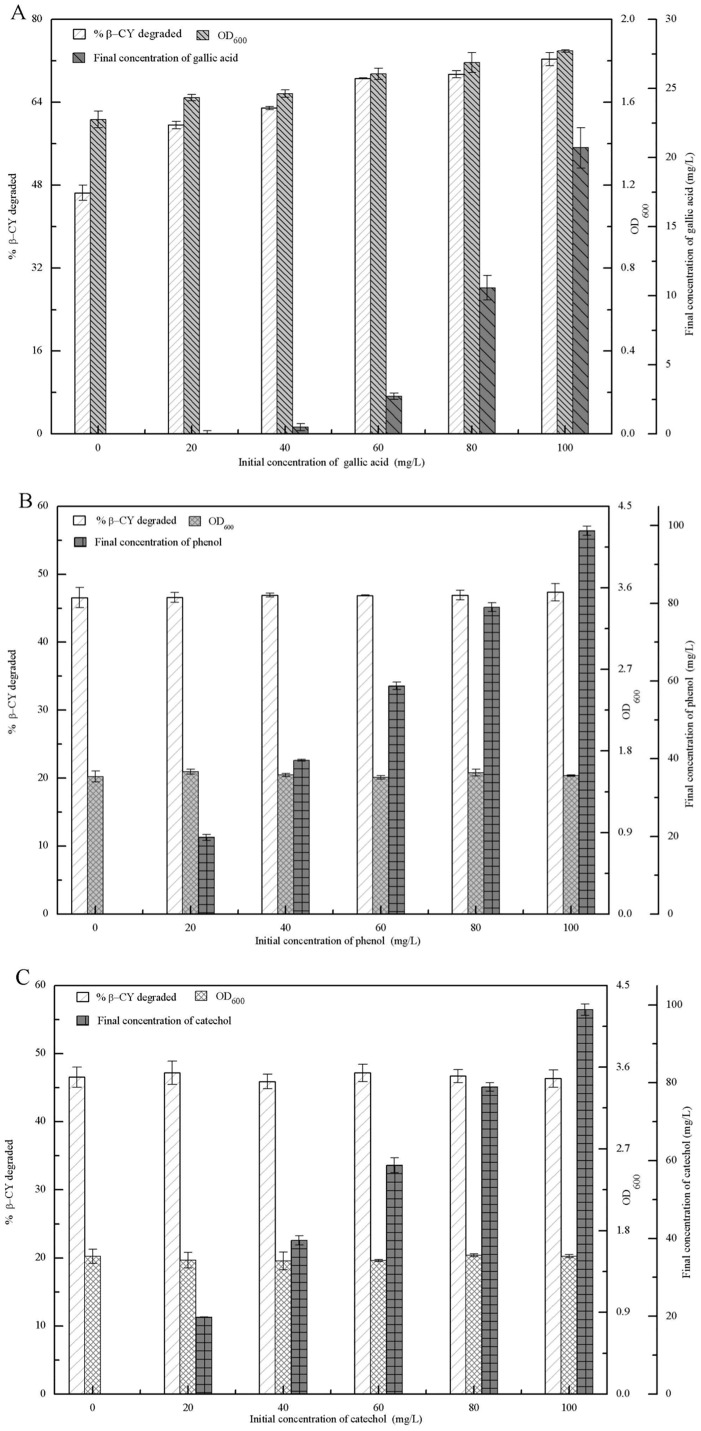
β-CY degradation, the biomass (OD_600_) of strain B-1, and the final 3-PBA-metabolite concentration in LB-MS medium supplemented with gallic acid, phenol, or catechol after 72 h. (A) gallic acid, (B) phenol. (C) catechol.

β-CY degradation, 3-PBA-metabolite concentrations, and the biomass of strain B-1 were investigated in the presence of various concentrations of gallic acid, phenol, or catechol after 72 h. As illustrated in Fig 5A, the final concentration of gallic acid was lower than its initial concentration. When gallic acid concentration rose from 20 mg/L to 100 mg/L, β-CY degradation and the optical density (OD_600_) values of strain B-1 increased from 46.54% to 72.34% and 1.52 to 1.80, respectively. These results indicated that strain B-1 was able to use gallic acid as a carbon or energy source for β-CY degradation and cell growth. As shown in Fig 5B and 5C, no changes was observed in β-CY degradation or optical density (OD_600_) values when the concentrations of phenol and catechol increased from 20 mg/L to 100 mg/L. Compared with the initial concentrations of phenol and catechol, their final concentrations did not significantly decrease. These results verified that phenol and catechol could not be utilized by strain B-1 and that they did not affect β-CY degradation or cell growth. Therefore, phenol and catechol did not affect 3-PBA degradation by strain M-4, and gallic acid, the toxic intermediate from co-metabolic degradation of 3-PBA, improved β-CY degradation by strain B-1.

## Discussion

Organic contaminants and their associated metabolites can be efficiently degraded in co-cultures of microbes via co-metabolism [16]. Here, co-culture of strains B-1 and M-4 was more efficient than strain B-1 alone in degrading β-CY (Fig 1). This is primarily due to that strain B-1 is unable to degrade 3-PBA, with high concentrations of 3-PBA capable of inhibiting β-CY degradation (Fig 2B). β-CY degradation and 3-PBA concentration after co-culture of strains B-1 and M-4 were higher and lower, respectively, than those resulting from co-culture of *B*. *licheniformis* B-1 and *Sphingomonas* sp. SC-1 [13]. Moreover, the half-life (*t*_1/2_) of β-CY and the 3-PBA yield coefficient (*Y*) from the co-culture of strains B-1 and M-4 were also shorter and lower, respectively, than those from co-culture of strains B-1 and SC-1 [13]. These results indicated that microbial co-culture efficiently degraded β-CY and 3-PBA via co-metabolism.

The co-metabolic degradation of organic contaminants is usually inhibited by toxic intermediates [15, 16]. In this study, the intermediate metabolites from co-metabolic degradation of β-CY by strain B-1 were chrysanthemic acid and 3-PBA. Chrysanthemic acid did not affect β-CY degradation or the growth of strain B-1 (Fig 2A). Deng et al [19] also found that chrysanthemic acid was unable to support the growth of *Aspergillus niger* YAT or enhance β-CY degradation. Additionally, 3-PBA exerted a toxic inhibition effect on β-CY degradation and the growth of strain B-1 (Fig 2B) and constituted the toxic intermediate of co-metabolic β-CY degradation by strain B-1. This was due to the inhibition of key enzymes involved in co-metabolic degradation [15, 18]. Liu et al [13] also reported that 3-PBA inhibited the co-metabolic degradation of β-CY and the growth of β-CY-degrading strain, and the possible reason was that 3-PBA directly reduced the expression of key enzyme from the gene transcriptional level and the toxic inhibition exerted directly on key enzyme could inactivate the metabolic pathways and directly stop the co-metabolic degradation [15].

Moreover, the intermediate metabolites from co-metabolic degradation of 3-PBA by strain M-4 were gallic acid, phenol, and catechol. Phenol and catechol can improve the degradation of 3-PBA and enhance the growth of strain M-4 (Fig 3B and 3C). Deng et al [19] also reported that *A*. *niger* YAT efficiently degraded phenol and catechol. This is due to fungi exhibiting strong oxidative/reductive capability supporting transformation of phenolic compounds into straight-chain olefin acids by dioxygenases and hydroxylases, and the subsequent ability to use straight-chain olefin acid as a carbon source [22, 23]. Gallic acid is the toxic intermediate of co-metabolic degradation of 3-PBA by strain M-4 and inhibited 3-PBA degradation and the growth of strain M-4 (Fig 3A). Kang et al [24] also reported that gallic acid inhibited microbial growth, and the mechanism of action was inhibition of extracellular microbial enzymes required for microbial growth or direct action on microbial metabolism through inhibition of oxidative phosphorylation [25].

The co-metabolic degradation of β-CY (or 3-PBA) was also affected by intermediate metabolites from co-metabolic degradation of 3-PBA (or β-CY). Phenol and catechol did not affect the degradation of β-CY or the growth of strain B-1, while gallic acid improved both of them (Fig 5). This may be because strain B-1 was able to utilize low concentrations of gallic acid through the activity of gallic acid decarboxylase [25]. Additionally, Chrysanthemic acid showed no effect on co-metabolic degradation of 3-PBA or the growth of strain M-4, while 3-PBA promoted cell growth. 3-PBA was capable of improving the growth of *Sphingomonas* sp. SC-1, *Pseudomonas pseudoalcaligenes* POB310, and *Bacillus thuringiensis* ZS-19, because they were capable of using 3-PBA as a carbon or energy source [26–28]. However, strain M-4 degraded 3-PBA via co-metabolism, but was unable to utilize 3-PBA as a carbon or energy source [15]. Our findings suggested that the 3-PBA metabolites phenol and catechol can support the growth of strain M-4 (Fig 3B and 3C).

**Fig 6:**
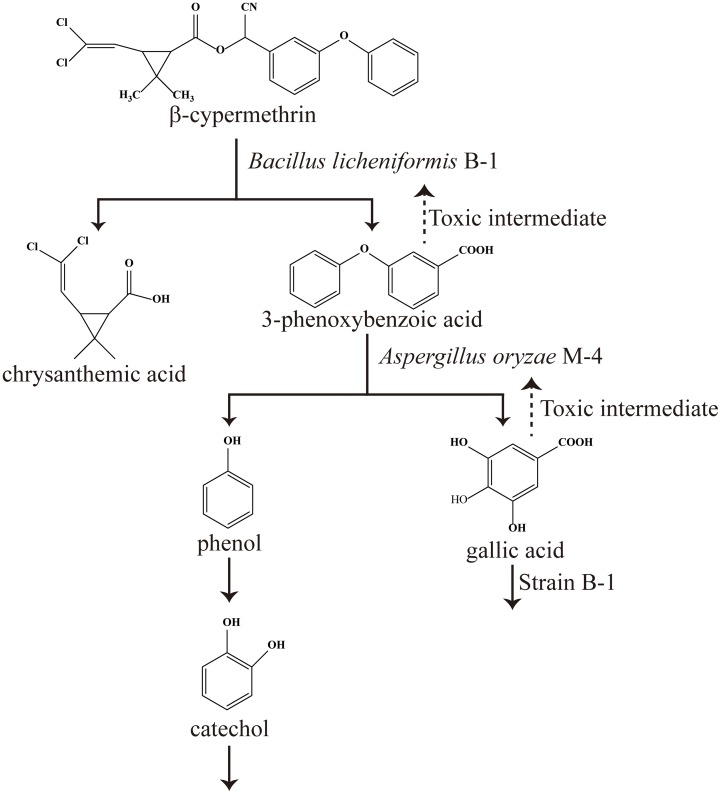
Degradation pathways of β-CY and 3-PBA by co-culture of strains B-1 and M-4.

The degradation pathways of β-CY and 3-PBA by co-culture of strains B-1 and M-4 were shown in Fig 6. 3-PBA and gallic acid were the toxic intermediate from co-metabolic degradation of β-CY and 3-PBA, respectively (Fig 6). We concluded that β-CY was efficiently degraded by strain B-1 based on strain M-4 removal of the toxic intermediate (3-PBA) from co-metabolic degradation of β-CY and production of gallic acid from co-metabolic degradation of 3-PBA during co-culture of strains B-1 and M-4 (Fig 6). Similarly, 3-PBA was efficiently degraded by strain M-4, because strain B-1 removed the toxic intermediate (gallic acid) from co-metabolic degradation of 3-PBA (Fig 6).

## Conclusions

The co-culture of strains B-1 and M-4 degraded β-CY efficiently and 3-PBA completely. 3-PBA, the toxic intermediate from co-metabolic degradation of β-CY, inhibited β-CY degradation and the growth of strain B-1. Additionally, gallic acid, the toxic intermediate from co-metabolic degradation of 3-PBA, inhibited 3-PBA degradation and the growth of strain M-4. Our findings indicated that strains B-1 and M-4 efficiently degraded gallic acid and 3-PBA, respectively. Therefore, the mechanism for efficient degradation of β-CY and 3-PBA by co-culture of strains B-1 and M-4 involved the toxic intermediate from co-metabolic degradation of β-CY (or 3-PBA) being efficiently degraded by strain M-4 (or strain B-1).

## Supporting Information

S1 Dataset Dates of the figures and table. (XLSX)
